# Temporary Exclusion of Cattle from a Riparian Zone Using Virtual Fencing Technology

**DOI:** 10.3390/ani9010005

**Published:** 2018-12-22

**Authors:** Dana L. M. Campbell, Sally J. Haynes, Jim M. Lea, William J. Farrer, Caroline Lee

**Affiliations:** 1 Agriculture and Food, Commonwealth Scientific and Industrial Research Organisation, New England Highway, Armidale, NSW 2350, Australia; jim.lea@csiro.au (J.M.L.); caroline.lee@csiro.au (C.L.); 2 Adjunct to School of Environmental and Rural Science, University of New England, Armidale, NSW 2351, Australia; 3 Agersens, Pty Ltd., Melbourne, VIC 3000, Australia; shaynes@agersens.com (S.J.H.); will.farrer@agersens.com (W.J.F.)

**Keywords:** commercial, GPS, group behaviour, associative learning, automated technology, welfare

## Abstract

**Simple Summary:**

Cattle can help to graze riparian zones when managed effectively. Virtual fencing technology, where cattle wear collar devices that provide audio followed by electrical signals around a GPS-based fence, could be used in areas that are difficult to physically fence. An early experimental automated collar device prototype was tested in excluding 10 cattle from a riparian zone in Australia. Animals were given free access to an 11.33-hectare area for three weeks, excluded from river access by a virtual fence for ten days (2.86-hectare inclusion zone), followed by free access again for six days. Animals were almost exclusively contained by the virtual fence. All animals approached the virtual fence over the trial duration and received both audio cues and electrical stimuli, but individual animals differed in how often they tested the virtual boundary. Over time, animals learned to respond to the audio cue alone to avoid receiving an electrical stimulus. Following fence deactivation all animals re-entered the previously excluded area. Further research with more groups and longer periods of exclusion using updated collar devices would determine the scope of virtual fencing technology for cattle grazing control.

**Abstract:**

Grazing cattle can both negatively and positively impact riparian zones, dependent on controlled grazing management. Virtual fencing technology, using collar devices that operate via GPS can provide audio cues and electrical stimuli to temporarily exclude cattle from specified areas as desired. An early experimental prototype automated virtual fencing system was tested in excluding ten cattle from a riparian zone in Australia. Animals were given free access to an 11.33-hectare area for three weeks, excluded from river access by a virtual fence for ten days (2.86-hectare inclusion zone), followed by free access again for six days. Animals were almost exclusively contained by the virtual fence. All animals received audio cues and electrical stimuli with daily fence interactions, but there was high individual variation with some animals first approaching the fence more often than others. Overall, there was an approximately 25% probability that animals would receive an electrical stimulus following an audio cue. Individual associative learning may have been socially-facilitated by the group’s behaviour. Following fence deactivation, all animals re-entered the previously excluded area. Further research with more groups and longer periods of exclusion using updated collar devices would determine the scope of virtual fencing technology for cattle grazing control.

## 1. Introduction

Grazing cattle can have detrimental effects on riparian zones (the interface between land and rivers) through reduction of water quality [1], destruction of habitats for native animals [1,2,3], and reduced species diversity [4]. When managed effectively, cattle can also have constructive effects through grazing of more dominant and/or invasive vegetation that then promotes riparian species diversity [5,6]. Virtual fences―providing boundaries without physical structures―thus represent an opportunity for improved management of cattle around riparian zones and other environmentally sensitive areas where the implementation and maintenance of physical fencing is not possible. Virtual fences may be used to both exclude cattle from areas that are difficult to fence, and/or to temporarily exclude cattle as desired. 

While there have been several decades of research into various types of virtual fencing technology, to date, there are few commercially available products [7]. Patented virtual fencing technology developed by the Commonwealth Scientific and Industrial Research Organisation (CSIRO [8,9]) operates by pairing a neutral stimulus (an audio tone) with an aversive stimulus (an electrical pulse). Associative learning over time results in the animal being able to control the number of electrical stimuli received by responding to the audio cue alone. An early experimental prototype developed by Agersens Pty Ltd based on the CSIRO-patented algorithm that tracks animal location and movement via GPS and emits audio cues and electrical stimuli relative to a virtual (GPS) fence was used in this experiment. 

Studies conducted with these early automated virtual fencing collar prototypes in experimental field settings demonstrated that, over time, cattle were able to respond to an audio cue alone [10,11]. However, individually-tested animals did show wide variation in both learning rate and behavioural responses to the cues [11]. These included undesirable responses such as running forward and learning the location of the fence line, thus avoiding any subsequent interaction with it [11]. Location-learning of fence lines would limit the use of virtual fencing technology for temporary exclusion. However, individual testing is a more artificial scenario for cattle and may be expected to elicit different behavioural responses and learning curves compared to a group setting. Cattle in groups are driven to stay with the herd, they form social relationships, and social facilitation may influence behaviour [12,13]. Subsequent group testing with the same individually-tested cattle showed that virtual fences were able to successfully restrict cattle movement as intended, and that within a few hours of fence lines shifting, animals moved into newly accessible areas [10]. This indicated they were responding to the audio cue rather than learning a fence location but there was still variation in individual associative learning rates [10]. Knowledge of how cattle in a group setting associatively learn to respond to the audio cue to avoid the electrical stimulus is limited, including whether all individuals have equal interactions with the virtual fence. The ability of all animals to learn to respond to the audio cue is important to ensure welfare outcomes are acceptable, as described in a recent welfare assessment framework for learning centred around the controllability and predictability of stimuli/new technology [14]. To date, there have been no commercial trials conducted with these automated virtual fencing collar prototypes to determine their application for temporarily excluding cattle from an environmentally-sensitive riparian zone. 

The first objective of the current study was to determine if automated virtual fencing collar prototypes could exclude a group of ten cattle from a riparian zone on a commercial cattle property in New South Wales (NSW), Australia. The second objective was to determine if cattle would learn to respond to the audio cue alone and whether there was variation between individuals and their interactions with the virtual line. The third objective was to assess whether animals would return to the previously excluded riparian zone when the virtual fence line was removed. 

## 2. Materials and Methods 

### 2.1. Ethical Statement

The experiment was approved by the CSIRO FD McMaster Laboratory Chiswick Animal Ethics Committee (AEC17/08) prior to the start of the experimental period.

### 2.2. Animals and Baseline Experimental Protocol

**Figure 1:**
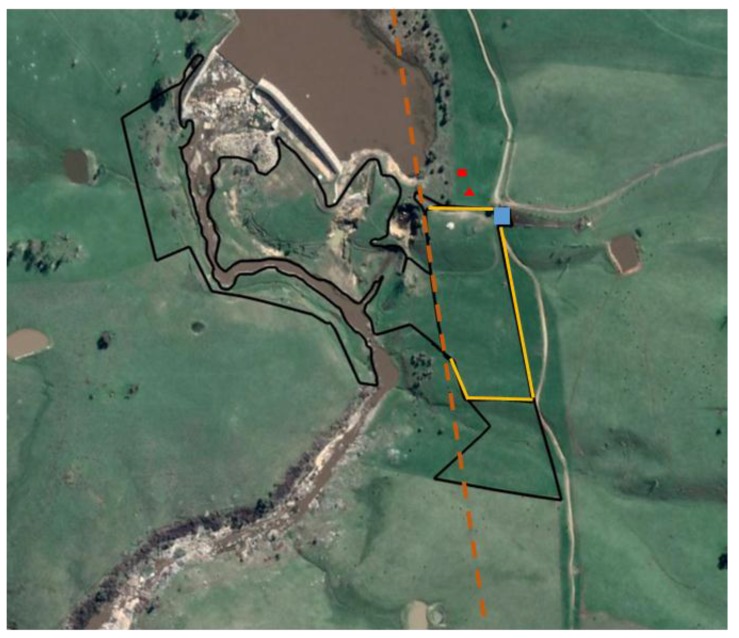
A satellite image (Google Earth^®^) of the commercial riparian zone. The dark tracks indicate the area available to the cattle as constrained by physical fences or topography. The dashed orange line indicates the single virtual fence line implemented for ten days. The solid yellow lines indicate the inclusion zone that the cattle had access to when the virtual fence line was in place and the blue square indicates the position of the water trough when river access was excluded. The red triangle indicates the position of the base station, and the red square indicates the position of the observers.

Eleven naïve (to virtual fencing technology) Angus heifers (approximately 400–450 kg in weight, 30 months of age) were initially used in the May 2017 23-day experimental trial based on a commercial cattle property near Tumbarumba in NSW, Australia. Group size was determined by the available number of prototype devices; 11 individuals is also similar to the mean group size of 10.5 animals observed in feral cattle populations [12]. Only ten animals remained for the entire trial duration due to a collar malfunctioning, with further details provided in Section 2.4. Two weeks prior to placement of the experimental prototype automated virtual fencing collar device (eShepherd™, Agersens Pty Ltd, Melbourne, VIC, Australia) that commenced data collection, all cattle were fitted with MooMonitor^®^ collar straps (Dairymaster, Causeway, County Kerry, Ireland) including approximately 1.5 kg of hanging collar counterweights and provided first access to the experimental riparian zone paddock area (total paddock area approximately 11.33 hectares, Figure 1). Following two weeks of acclimation to the collars and paddock, the experimental prototype automated virtual fencing collar device (approximately 0.8 kg in weight, 19 cm length × 10 cm width × 5 cm height) was fitted onto the MooMonitor^®^ collar straps and sat on the side of the animal’s neck. All animals were released back into the paddock area and 23 days of data collection for the trial began. The collar devices initially collected GPS-based data on use of the paddock area but no virtual fence line was set. MooMonitor^®^ devices were also in place on the collars throughout the trial duration but, unfortunately, raw MooMonitor^®^ data were unable to subsequently be obtained due to commercial conflicts of interest and thus were not included in any analyses.

### 2.3. Virtual Fencing Collar Device

The experimental prototype automated virtual fencing collar device (eShepherd^TM^, Agersens Pty Ltd, Melbourne, VIC, Australia) used GPS technology to monitor the animal’s movement including a real-time measure of the animal’s speed, direction, and position. GPS coordinates were used to specify a virtual fence boundary that was transmitted to the collar via a radio frequency link. As the animal reached the virtual fence line, the collar device emitted a distinctive but non-aversive audio tone within the animal’s hearing range (for further details, see [11]. If the animal stood still or turned away, no electrical stimulus was applied. If the animal continued walking towards the virtual fence, a short sharp pulse train in the kilovolt range was immediately delivered (values are commercial in confidence). This audio cue/electrical stimulus sequence was repeated continuously if the animal continued further into the “exclusion zone”, i.e., in this instance, towards the river. If the animal walked back out of the exclusion zone, no stimuli were applied. Stimuli were not applied if the animal was detected as moving over a specified maximum velocity (e.g., running) and deemed to be unresponsive. If an individual animal received a specified number of stimuli within a specified time frame, the device entered standby mode and stimuli were not applied for a specified time frame (values are commercial in confidence). Finally, the collar also included a “grazing algorithm”. The natural behavioural pattern of grazing can mimic the correct response by the animal to the collar cues of movement forward and stopping at an audio cue. Therefore, if an animal gradually encroached on the exclusion zone by grazing, after three consecutive audio cues while slowly moving forward paired with stopping, an electrical stimulus was applied (the three consecutive audio cues followed by an electrical stimulus is referred to as the grazing algorithm henceforth). The date, time, GPS location of the animals and any cues delivered were logged by the collars to be downloaded for analysis. All collars reported to a base station located adjacent to the experimental paddock (Figure 1) that enabled viewing of the collar data online in real time.

### 2.4. Virtual Fence Line Experimental Protocol

Following seven days of collecting baseline GPS data on the paddock areas that the animals accessed, a straight virtual fence line was set in front of the riparian zone within the paddock creating an “inclusion zone” (2.86 hectares) where cattle could graze and access a water trough, and an “exclusion zone” that included the river area and land accessed by crossing the river (Figure 1). The experienced producer at the commercial property visually determined that there was sufficient pasture within this smaller area to feed all the animals across the fence activation period but no specific pasture measurements were taken during the trial. Water was available via a trough within the inclusion zone (Figure 1). An electric fence at one end of the paddock also had to be shifted down to avoid creating an additional small exclusion zone with the unevenly-shaped inclusion zone paddock and straight virtual fence line (Figure 1). All animals were observed for the first interactions with the fence line to ensure there were no extreme adverse reactions such as bolting through the virtual line or into physical fences, circling, vocalising or bucking. The animals were then left in the inclusion zone for approximately ten days from the afternoon of Day 1 through to the afternoon of Day 11. However, on Day 10, a pilot herding trial occurred within the inclusion zone as part of a separate dataset not reported on here and, thus, no data from this day (GPS location or collar cue data) were included in the current analyses. This pilot herding trial assessed whether animals could be moved across the width of the inclusion zone by setting a single fence at the location cattle were desired to be moved to and placing electric tape across the riparian zone. The animals did not cross into the riparian exclusion zone during this pilot trial. After ten days of activation, the virtual fence line was turned off and GPS data were recorded for a further six days to assess animal movement. On the day prior to fence deactivation, one animal (5) was removed from the trial as there was believed to have been a functionality error in the delivery of the electrical stimulus with inconsistent strength in the electrical pulse. Following the pilot herding trial, all animals had 8 h with the previous fence activated again and were observed to interact with this fence that prevented access to the river prior to it being deactivated. This ensured the animals were still responding to the virtual fence following the pilot herding trial and, thus, this testing day was determined to have had minimal effect on the animals’ subsequent movement behaviour.

Cattle were regularly monitored both by observers present at the site or online remotely. When the fence was activated, observers were present on site across all daylight hours, prior to activation, and following deactivation, observers were present on site for approximately 1 h, and also confirmed collar functionality online remotely every 2–3 h across the day. Throughout the trial, the batteries on the experimental prototype virtual fencing devices had to be recharged approximately every three days. All animals were brought into the yards, collars removed in the crush, batteries changed out, collars refitted with charged batteries and animals released back into the paddock. This process typically took approximately 2–3 h and data were not recorded during these periods. 

### 2.5. Data and Statistical Analyses

All GPS positional data for each animal were compiled for visual display of their location prior to, during the virtual fence line, and then following fence deactivation. All audio and electrical stimuli emitted per day for each individual animal were compiled for visual display. Animal 5 was removed from this and the following datasets as there was a functionality error in the delivery of the electrical stimulus. Associative learning of the animal group was analysed with logistic curves as previously used in [11,15]. For each animal (i), the sequence of audio and electrical stimulus events was summarised as a paired variable; the audio event number Xij and a binary variable Zij, which was zero if the fence interaction event was an audio-only cue (Xij) or one if the fence interaction event was an audio and electrical stimulus paired together, as described in [11,15]. The paired variables for 10 individuals were analysed by fitting a logistic curve to the data using the non-linear least squares function in the statistical software package R (The R Development Core Team, Version 3.3.3, Vienna, Austria). A general logistic curve (or Richards curve [16]) of the form

$$\pi =a+\frac{c}{1+\exp(-b(x-m))}$$

was fitted where *π* is the probability that *Zi* = 1, *a* is the lower asymptote, *a + c* is the upper asymptote, *b* is a slope parameter, and *m* is the point of inflection. Two logistic curves were fitted to the data. The dataset for the first curve included all stimuli received by the animals across the trial duration. The dataset for the second curve had the first two audio cues of the grazing algorithm removed. Although the collar records do not specifically state when the grazing algorithm was applied, any sequence of three audio cues followed by an electrical stimulus that occurred within 1 min, was interpreted as likely being the grazing algorithm and the first two audio cues were removed (this occurred 53 times). 

In this study, the results were interpreted in relation to animal behaviour. The slope parameter is related to the rate at which behaviour changes, with a negative slope parameter indicative of the proportion of animals receiving the electrical stimulus decreasing with repeated audio events. The upper asymptote in a negative slope parameter is the proportion of naïve animals receiving an electrical stimulus, while the lower asymptote is the proportion of animals receiving an electrical stimulus across learning. The point of inflection is the audio event or fence interaction at which half of the change in proportion from upper to lower asymptote has occurred. It would be predicted that, when the audio cue and electrical stimulus are novel, all animals would receive both (proportion of electrical stimulus applied would be equal to 1), but this would reduce with increasing fence interactions as animals learn to respond to the audio cue alone. In fitting the curve, no constraints were applied, thus allowing the asymptotes to be outside the meaningful range of 0–1, and the slope parameter to be >0.

Finally, separate groups of fence interactions were also summed across the fence activation period. These were defined as a minimum of 10 min between when one individual received a cue, and the next individual received a cue. If greater than 10 min had elapsed between individuals testing the fence line, they were classified as separate group interactions. If multiple animals tested the fence line and received cues following the first individual in less than 10 min, these were all considered as one group of interactions. For example, if one animal tested the fence, after 5 min a second animal tested the fence, after another 5 min a third animal tested the fence and so forth, these were all still classed as a single group interaction until a minimum of 10 min had elapsed between individuals receiving fence cues. The identity of the first individual to interact with the fence/receive an audio cue was counted to tabulate the percentage of separate interactions where specific individuals were the first to interact. Animal 5 was included in this dataset because this was aimed at looking at interactions of the group. Exclusion of Animal 5 would provide misleading data on the frequency that each individual in the group was first to interact. 

## 3. Results

**Figure 2:**
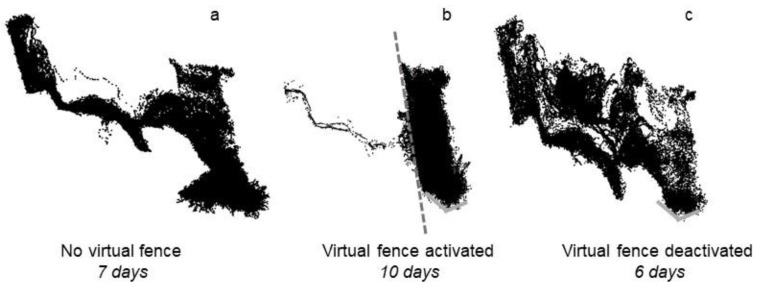
The GPS locations of all animals in the virtual fencing trial on a commercial riparian zone property. Images display cattle movement: (**a**) when no virtual fence was present; (**b**) when a virtual fence was activated (dashed line); and (**c**) when that virtual fence was subsequently deactivated with days of each period length indicated. Solid grey lines indicate a physical electric fence that was placed to remove a corner zone of the paddock that would have been logistically difficult for the cattle once the virtual fence was activated.

All animals were successfully contained within the exclusion zone following activation of the fence for the majority of the exclusion period (Figure 2b). As indicated in Figure 2b, animals did cross over the fence line into the exclusion zone on multiple occasions, but did not travel far into the exclusion zone. The exception was three days after the fence was activated when four animals (Animals 5, 6, 11, and 14) crossed into the exclusion zone for approximately 30 min (Figure 2b). As per the device description (see Methods), the collars entered a standby mode once a specified number of consecutive electrical stimuli had been received. Following the cessation of this safety feature, the collars provided both audio and electrical cues again to turn the animals back towards the inclusion zone where their herd mates were located. When animals were moving back towards the inclusion zone (walking or grazing), as per the collar algorithm, they did not receive any stimuli. Following deactivation of the fence, within approximately 2 h, all animals crossed back into the previous exclusion zone and accessed the full area available to them, including additional area to what was accessed prior to the fence being activated (Figure 2a,c). 

**Figure 3:**
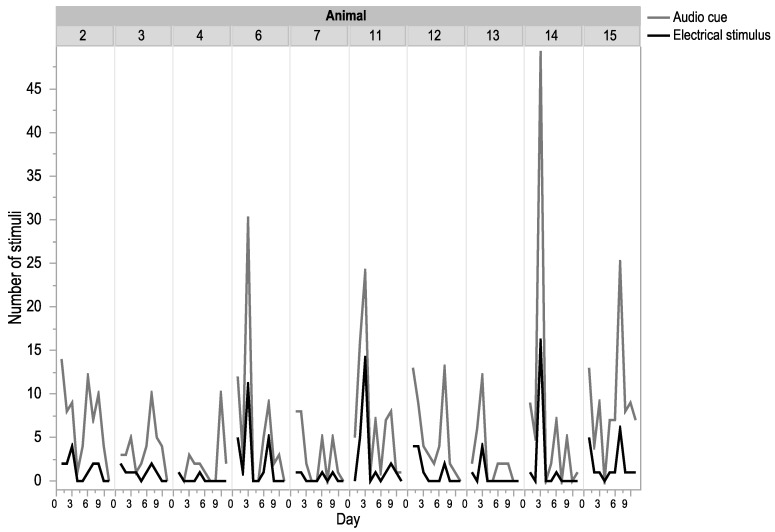
The number of audio and electrical stimuli that each individual animal received during the 10-day period of virtual fence activation. Animal 5 was removed from this dataset as there was a functionality error in the delivery of the electrical stimulus.

All animals interacted with the fence and received both audio and electrical stimuli cues but there was high individual variation in both the number of fence interactions and the ratio between audio cues and electrical stimuli (Figure 3). For example, Animal 14 received over 45 audio cues on a single day, whereas Animal 7 never received over 10 on any given day (Figure 3).

**Figure 4:**
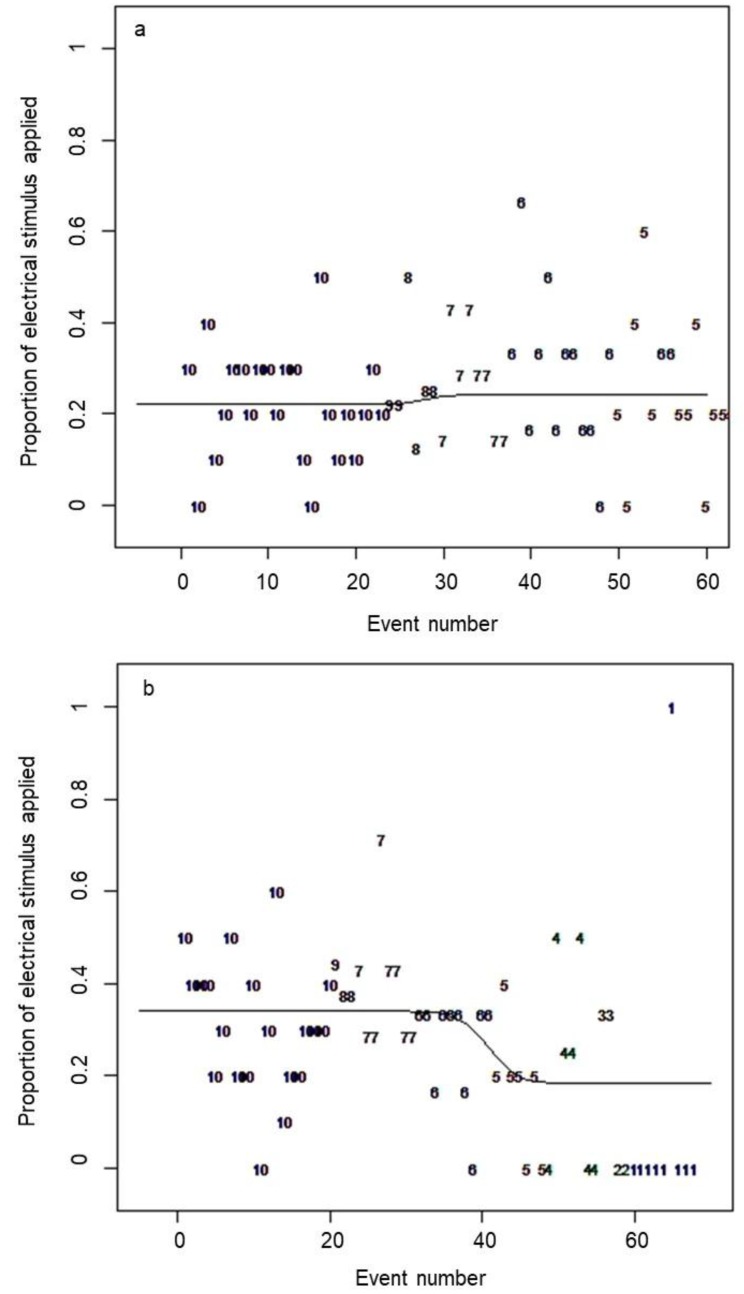
The logistic learning curve for: (**a**) all animals across all combined days; and (**b**) all animals across all combined days but with the grazing algorithm’s two additional audio cues removed. The *y*-axis is the proportion of animals testing the fence line that received an electrical stimulus and the *x*-axis is the number of individual events or interactions with the fence line (i.e., one animal receiving a single cue is a single event). The numerals are the number of animals that tested the fence line at each event number (i.e., (**a**) all ten animals interacted with the fence at least once, but only five animals interacted 60 times or more). Animal 5 was removed from these datasets as there was a functionality error in the delivery of the electrical stimulus negating accurate determination of associative learning. Across all events, approximately 25% of animals received an electrical stimulus.

Estimated parameters for the logistic curves generated in R are presented in Table 1. In the first analysis that used all cues received by the animals, a relatively straight line was generated rather than a clear curve as in previous virtual fencing trials that have applied the same analysis to the associative learning of the animal group [11,15]. In previous results, the upper asymptote is close to 1, reflecting the initial learning of the animals when they do not yet know the association between the audio cue and electrical stimulus and the presence of the virtual line. The curve drops over increasing numbers of interactions as the animals learn to respond to the audio cue alone [11,15]. However, in this study, the first analysis that included all cues received generated a comparatively straight line with no significant difference between the upper and lower asymptotes, indicating a low probability of an animal receiving an electrical stimulus following the audio cue across the trial duration (approximately 25% probability as determined by the placement of the fitted line, Figure 4a). When the first two audio cues of the grazing algorithm were removed, there was a higher probability of receiving a shock initially (approximately 35% probability, Figure 4b).

**Figure 5:**
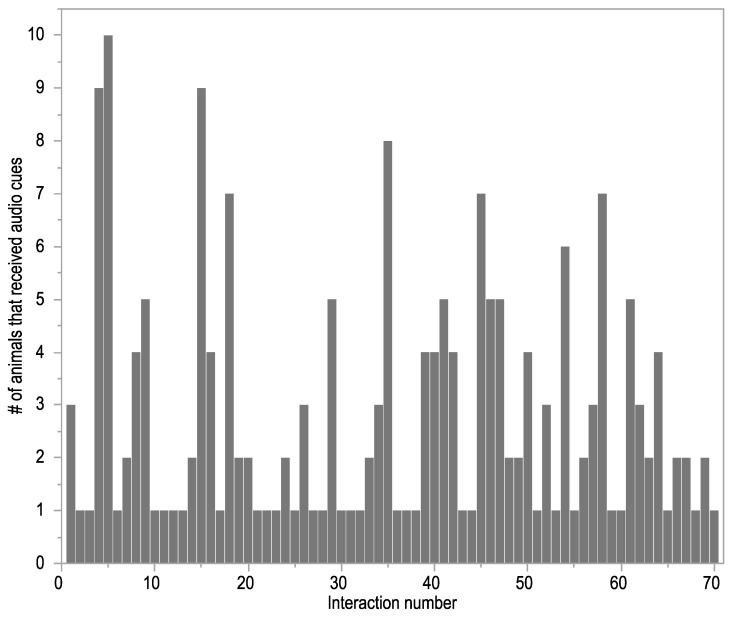
The number of animals that received an audio cue at each separate group of interactions with the virtual fence across a 10-day period of fence activation. Separate groups of interactions by the same or different individuals occurred at least 10 min apart.

A total of 70 separate groups of interactions occurred with the virtual fence over the activation period. Not every animal interacted with the fence within the same time interval. For example, on the first interaction with the fence, only three animals received audio cues and, on many subsequent occasions, it was only one animal that received an audio cue (Figure 5). Live observations of the first fence interaction showed that, although only a few animals received cues, the whole group was turned away. In this instance, one animal received an electrical stimulus and her reaction caused the entire group to retreat. There was also variation in which individual animal was first to interact (i.e., receive an audio cue) with the fence across the separate interaction periods (Table 2). All animals were first on at least one occasion but two individuals represented approximately one third of all first interactions (Table 2). 

## 4. Discussion

This study showed that a small group of ten cattle could be temporarily excluded from a riparian zone on a commercial property in Australia, using an early experimental prototype of an automated virtual fencing collar. Across the virtual fence activation period, all animals interacted with the fence and received collar cues, but the number of fence interactions, number of audio cues and electrical stimuli received varied between individuals. Across the 10-day virtual fence exclusion period, the probability of receiving an electrical stimulus was low, indicating animals were associatively learning the pairing between the audio cue and electrical stimuli. Their learning potentially could have also been socially-facilitated by observing other individuals.

In previous experimental trials using the same early experimental collar prototypes, individual animals displayed variation in associative learning rate both when tested individually [11], and in a small group [10]. All animals in the paddock setting in the previous trial showed a clear pattern of increasing responses to the audio cue alone thus avoiding a subsequent electrical stimulus [10]. In contrast, in this study, animals were appropriately responding to the audio cue alone, and, thus avoiding a subsequent electrical stimulus starting from the first time they heard the audio cue (see lack of a clear learning curve in Figure 4a). Studies in sheep report a similar finding of animals responding to the audio cue on initial interactions with the fence both when tested individually [17] or in a group [18]. This could result from two factors. Firstly, the learning curve is likely influenced by the grazing algorithm that was applied if animals slowly grazed into the fence line versus walking into it and did not turn away at the first audio cues. Thus, animals were recorded as appropriately responding to the audio cue by stopping as part of the natural grazing pattern. When the additional audio cues of the grazing algorithm were removed from the analyses, the learning curve showed a higher probability of receiving an electrical stimulus initially, but this decreased over time. Secondly, it is hypothesized that animals may have been socially facilitated in their learning and were responding to the audio cue based on observations of other animal’s behaviour. This type of response was observed during the first fence interactions where an adverse reaction from one individual that received an electrical stimulus resulted in the whole group turning back from the fence. However, the role of social facilitation was not formally assessed and thus would be valuable to investigate in further studies. Additionally, the influence of individuals that may not learn or that may not have a proper functioning collar (as per Animal 5 in this study) needs to be understood. Herd social attraction may minimize the impacts of potential collar malfunction in large group sizes but this remains to be assessed. The low probability of receiving an electrical stimulus across the trial, indicates that the stimuli are predictable and controllable which is likely to lead to an acceptable welfare outcome for the cattle [14]. However, further investigation into the relative roles of individual associative and socially-facilitated learning would clarify if group behaviour has positive or negative impacts on learning rate. This could include physiological measures such as heart rate to evaluate the emotionally positive outcomes of learning for the animals [19]. Additionally, this was only a small group of cattle and it is not known whether all animals in a larger group would learn the association between cues. 

All individuals were first to approach the fence on at least one occasion, but specific individuals were more likely to be the first to interact than others. There was no distinct single leader of the group specifically in terms of first access to the fence, but two individuals comprised over one third of all first interactions. One of these animals was the individual that was eventually removed due to a detected collar fault in the consistency of the strength of the electrical stimulus delivery. She may have been more likely to test the fence boundary due to inconsistent stimulus delivery resulting in poor associative learning. However, it could also be that more dominant individuals within the group are more likely to test a virtual line. This is similar to other studies that have shown dominant individuals will influence the general herd movement patterns [20]. Evidence for leaders in cattle groups and their influence on the group’s movements is inconsistent [20,21,22,23]. Both learning of the signals and first movements towards the fence could be influenced by social relationships between individuals within a group [22], an avenue that warrants further investigation. The evidence from this study does suggest that, as all animals did interact with the fence throughout the activation period, all animals would therefore need to be wearing collars rather than a proportion, or specifically selected individuals. This may differ for a large herd of cattle, but this remains to be tested.

The virtual fence excluded cattle from a water resource that they had regularly accessed previously for approximately ten days. The daily interactions with the fence, and the four animals that pushed through the fence on the third day, indicate the animals were still motivated to access the river area during the exclusion period but that the virtual fence was sufficient to deter them. Following deactivation of the fence, all animals accessed the previously excluded riparian area within approximately 2 h, including additional areas across the river that were not accessed prior to fence activation. It is not known whether this was a chance occurrence that the animals discovered a way to access new paddock areas, or whether it was a rebound effect of being restricted that motivated the animals to explore further. Dairy calves and heifers that were confined showed increased locomotor behaviour following release from confinement [24]. This same principle may apply to cattle who have been accustomed to larger roaming areas and have then had available paddock area significantly reduced. Behavioural time budget data could be used in future studies to determine if any differences were present in daily behavioural patterns of the animals (for example, standing, grazing, lying, and walking) prior to, during, and after fence activation. Minimal time budget differences were seen in cattle restricted with moving virtual fence lines [10], but the smaller area and exclusion from a resource that was previously accessed daily may have resulted in disruption of typical daily behaviours.

## 5. Conclusions

Overall, this study showed that early experimental prototypes of virtual fencing collar devices could temporarily exclude a group of ten cattle from a riparian zone and that animals re-entered the previously excluded area. Animals learned to respond to the audio cue alone, but this may have been socially-facilitated avoidance learning in addition to associative avoidance learning. Further studies would assess additional groups of animals using updated commercial collar devices that have refined delivery of the stimuli based on the animal’s behaviour. Longer periods of exclusion are necessary to understand the impacts and limitations of this technology, including long-term assessment of any welfare-related behavioural changes.

## Figures and Tables

**Table 1:** Estimated parameters for the logistic regression curves across combined days of the virtual fence activation period for all 10 individuals (Animal 5 was excluded due to errors in collar functionality) ^1^.

Dataset	Upper Asymptote	Lower Asymptote	Significance of Difference	Point of Inflection	Slope	Significance of Slope
**All interactions**	0.22	0.24	0.63	28.07	−1.05	0.96
**GA removed**	0.19	0.34	0.12	35.08	1.20	0.74

^1^ The first row included all interactions with the fence and the second row had the first two audio cues of each grazing algorithm (GA) removed. The upper asymptote indicates the proportion of events in naïve animals receiving an electrical stimulus following an audio cue upon reaching the fence. The lower asymptote indicates the proportion of animals that continue to receive an electrical stimulus on subsequent interactions with the fence. The difference between the asymptotes was tested for significance with α set at 0.05. The point of inflection indicates the mean number of attempts until half of the learning had occurred. The slope indicates the speed of transition from the upper to lower asymptote. The lack of a significant *p*-value in the slope indicates there was variation in the slope.

**Table 2:** The percentage of total separate groups of fence interactions (*n* = 70) in which a specific individual animal was the first to receive an audio cue, i.e., interact with the virtual fence.

Animal #	Percent First to Audio
2	15.7
3	8.6
4	7.2
5	17.1
6	4.3
7	1.4
11	12.9
12	5.7
13	4.3
14	5.7
15	17.1
